# BENEVOLENT AGEISM: THE CORRELATES OF OVERACCOMMODATION TOWARDS OLDER ADULTS

**DOI:** 10.1093/geroni/igz038.3518

**Published:** 2019-11-08

**Authors:** Jennifer F Sublett, Toni L Bisconti

**Affiliations:** The University of Akron, Akron, Ohio, United States

## Abstract

Using the Stereotype Content Model as a framework for understanding ageism, our two objectives are (1) examining the predictive utility of benevolent ageism on well-being outcomes and (2) identifying conditional relationships between sex, perceived age, benevolent ageism, and well-being outcomes. In a snowball sample of 150 older adults who were 65 years old and older, we examined sex, perceived age, ageism, environmental mastery, and depression. Our benevolent ageism scale is an expanded version of the Ambivalent Ageism Scale that included additional items of accommodation created by us. Environmental mastery and depression were assessed by standard, internally valid, measures. Using regression analyses, we found that benevolent ageism predicted depression above and beyond hostile ageism. Additionally, benevolent ageism uniquely predicted environmental mastery for men, whereas hostile ageism uniquely predicted environmental mastery and depression for women. Finally, perceived age was a better predictor of well-being than chronological age. It is essential to consider how benevolent ageism relates to well-being due to the tenets of the Stereotype Content Model. Additionally, delineating the ways that sex and perceived age contribute to double jeopardy vs. crisis competence in the face of benevolence will lead to a more intricate understanding of the paths in which overaccommodative behaviors relate to well-being in older adulthood.

